# Die COVID-19-Pandemie im Schuljahr 2020/21: Wie haben sich die kognitiven Grundfähigkeiten von Schulkindern der Primarstufe entwickelt?

**DOI:** 10.1007/s35834-022-00358-2

**Published:** 2022-09-20

**Authors:** Wolfram Rollett, Thomas Leitgeb, Katja Scharenberg

**Affiliations:** 1 Professur für Empirische Bildungsforschung mit dem Schwerpunkt Schulentwicklung, Pädagogische Hochschule Freiburg, Freiburg, Deutschland; 2 Zentrum für Digitale Kompetenz, Private Pädagogische Hochschule Burgenland, Eisenstadt, Österreich; 3 Professur für Bildungssoziologie, Pädagogische Hochschule Freiburg, Freiburg, Deutschland; 4 Pädagogische Hochschule Freiburg, Freiburg, Deutschland

**Keywords:** Kognitive Entwicklung, Primarstufe, Fernunterricht, Schereneffekte, COVID-19-Pandemie, Cognitive development, Primary school, Distance education, Achievement gaps, COVID-19 pandemic

## Abstract

Eine wichtige aktuelle Frage der empirischen Bildungsforschung ist, welchen Einfluss die zur Eindämmung der COVID-19-Pandemie getroffenen gesellschaftlichen Maßnahmen auf die kognitive Entwicklung von Schüler_innen hatten. Damit ist auch die Frage verbunden, ob bildungsbenachteiligte Gruppen von Schüler_innen in Abhängigkeit ihrer individuellen oder familiären Voraussetzungen besondere Nachteile in ihrer kognitiven Entwicklung erlitten haben. Um diesen Fragestellungen nachzugehen, wurden die Daten von 104 Schüler_innen der dritten Schulstufe dreier österreichischer Volksschulen (Primarstufe) hinsichtlich der Entwicklung ihrer kognitiven Grundfähigkeiten in den Bereichen *Schlussfolgerndes Denken* und *Rechnerisches Denken* (KFT 1–3; Heller und Geisler 1983) analysiert. Darüber hinaus wurden individuelle Merkmale der Schüler_innen sowie Merkmale ihres häuslichen Umfeldes erfasst. Die Testung der kognitiven Grundfähigkeiten erfolgte zu Beginn und Ende des Schuljahres 2020/21 und damit vor und nach den gesamtgesellschaftlichen und schulischen Maßnahmen zur Eindämmung der zweiten und dritten Corona-Welle in Österreich (u. a. Kontaktbeschränkungen, Schulschließungen, Fernunterricht und Quarantänemaßnahmen). Im Untersuchungszeitraum wäre nach metaanalytischen Befunden der Intelligenzforschung (Rindermann 2011) altersgemäß eine positive signifikante Entwicklung von Schüler_innen in den durchschnittlichen Testleistungen von etwa d = 0,40 zu erwarten gewesen. Empirisch zeigten sich jedoch keine signifikanten Veränderungen. Dies gilt auch für die Streuungen in den Testleistungen. Gleichzeitig entwickelten sich Kinder aus Elternhäusern mit höherem Bildungshintergrund und jene mit umfassenderer Ausstattung mit digitalen Endgeräten sowie mit Internetzugang signifikant besser. Die Befunde weisen damit darauf hin, dass sich die Kinder in unserer Stichprobe in dem durch pandemieeindämmende Maßnahmen geprägten Schuljahr im Durchschnitt nicht so positiv entwickelt haben, wie dies zu erwarten gewesen wäre. Dabei ist es auch zu Schereneffekten gekommen, die auf kognitive Einbußen bei benachteiligten Schülergruppen hinweisen.

## Abstract (en)

In current empirical educational research, one important question is how the societal measures taken to contain the COVID-19 pandemic affected students’ cognitive development. This also raises the question whether disadvantaged student groups suffered particular disadvantages in their cognitive development depending on their individual characteristics or family background. We analyzed a data set of 104 students of three Austrian elementary schools (Grade 3). Students’ basic cognitive abilities were assessed by two subscales (inductive reasoning, numerical thinking) of the Cognitive Abilities Test 1–3 (KFT 1–3; Heller and Geisler 1983). We also analyzed effects of students’ individual characteristics and indicators of their home learning environment. Basic cognitive abilities were assessed at the beginning and end of the 2020/21 school year and thus before and after societal and school-related measures to contain the second and third pandemic waves in Austria (including contact restrictions, school closures, distance learning and quarantine measures). According to intelligence research (Rindermann 2011), an age-appropriate development in students’ mean test performance of about d = 0.40 would have been expected over the nine-months study period. Empirically, however, there were no significant changes in the average and heterogeneity of students’ test performances. At the same time, children from homes with a higher parental educational background and those with more digital devices and internet access showed a significantly higher development. The findings thus indicate that, on average, the children did not develop as positively in the school year characterized by pandemic containment measures as would have been expected. Beyond this, our results indicate cognitive losses among disadvantaged student groups.

Pandemieeinschränkende Maßnahmen, die seit dem Frühjahr 2020 international ergriffen wurden (World Health Organization 2020), hatten weitreichende Auswirkungen auf die Lebenswelt von Kindern und Jugendlichen. Dies war u. a. bedingt durch generelle Kontaktbeschränkungen, Einschränkungen bei der Nutzung von Freizeitangeboten und -möglichkeiten, Quarantäneregelungen für Schüler_innen, einzelne Klassen oder ganze Schulen sowie allgemeine Schulschließungen. Individuelle Lern- und Erfahrungsmöglichkeiten waren hierdurch stark eingeschränkt und Schüler_innen verbrachten entsprechend sehr viel mehr Zeit im häuslichen Umfeld (Helm und Postlbauer 2021). Damit stellt sich die Frage, wie sich diese gravierenden pandemiebedingten Veränderungen in den Lebensverhältnissen von Kindern und Jugendlichen auf ihre Entwicklung ausgewirkt haben.

Für die Entwicklung schulischer Leistungen in Zeiten, in denen pandemieeinschränkende Maßnahmen getroffen wurden, sind generelle Entwicklungsnachteile für Schüler_innen sowie herkunftsbedingte Schereneffekte im Vergleich zu einem regulären Schuljahr für fachspezifische Schulleistungen in der Primar- und Sekundarstufe I empirisch bereits gut belegt (siehe unten). Für die Entwicklung der kognitiven Grundfähigkeiten bei Schulkindern fehlen derartige Analysen jedoch bisher.

Der vorliegende Betrag nimmt daher die kognitive Entwicklung österreichischer Schulkinder der dritten Klasse der Primarstufe über einen Zeitraum von neun Monaten in den Blick, der stark durch schulische und gesamtgesellschaftliche pandemieeinschränkende Maßnahmen geprägt war. Geprüft wird dabei, ob sich die untersuchten Kinder in ihren kognitiven Grundfähigkeiten in dem Maß positiv weiterentwickeln konnten, wie dies auch unter regulären Lebensbedingungen erwartbar gewesen wäre. Darüber hinaus wird Effekten potenziell bildungsbenachteiligender bzw. -bevorteilender Faktoren nachgegangen, die sich im Sinne von Schereneffekten auf die kognitive Entwicklung der Kinder auswirken können. Diese würden auf differentielle Entwicklungsverläufe in den kognitiven Grundfähigkeiten in Abhängigkeit von individuellen Merkmalen der Schüler_innen bzw. Indikatoren der sozioökonomischen und kulturellen Ressourcen der Herkunftsfamilien hinweisen.

## Theoretischer Hintergrund und Forschungsstand

Von den von der Politik zur Pandemiebekämpfung ergriffenen Maßnahmen waren Schulschließungen ohne Zweifel besonders einschneidende Ereignisse für Familien und ihre schulpflichtigen Kinder. In diesen Zeiten waren Familien nicht nur gefordert, die Betreuung ihrer Kinder zu gewährleisten, sondern sie auch beim schulischen Lernen zu unterstützen. Digitaler Fernunterricht erforderte von allen Beteiligten spezifische Kompetenzen, die zu Beginn der Schulschließungen weder bei Schüler_innen (Suter et al. 2021), den Eltern (Porsch und Porsch 2020) noch bei Lehrkräften (Huber et al. 2020) in einem für gelingende Lehr- und Lernprozesse immer im erforderlichen Ausmaß vorhanden waren. Auch die für das Lernen im Fernunterricht benötigte technische Ausstattung wurde überwiegend als eher unzureichend eingeschätzt (Lorenz et al. 2020). Aber selbst wenn Familien mit einem Internetzugang und den für das Lernen im Fernunterricht benötigten Endgeräten technisch ausgestattet waren, hatten Schüler_innen hierzu nicht immer den erforderlichen Zugang, da sie die Ressourcen z. B. mit Geschwistern oder den im Homeoffice arbeitenden Eltern teilen mussten (Huber et al. 2020, S. 48, 61). Gleichzeitig fehlte die sonst im Präsenzunterricht gegebene professionelle Begleitung bzw. Unterstützung durch die Lehrkräfte (van Ackeren et al. 2020). Die selbstständige Auseinandersetzung mit den schulischen Aufgabenstellungen wurde eine zentrale Herausforderung für die Schüler_innen. Dies gilt umso mehr für jüngere Schüler_innen, da sie entwicklungsbedingt über geringere kognitive und selbstregulatorische Ressourcen als ältere Schüler_innen verfügen (z. B. Lohaus und Glüer 2017).

Inzwischen liegt eine Reihe empirischer Studien zum Lernerfolg von Schüler_innen unter den Bedingungen der Pandemie vor. Diese zeigten u. a., dass sich die effektive Lernzeit im Fernunterricht im Vergleich zum regulären Schulunterricht bedeutsam verkürzt hat (Wößmann et al. 2021; Grätz und Lipps 2020). In Bezug auf den Lernerfolg kamen die meisten hierzu bisher vorliegenden nationalen und internationalen Studien zu dem Ergebnis, dass die Leistungsentwicklung der Schüler_innen insgesamt in den durch pandemieeinschränkende Maßnahmen geprägten Schuljahren beeinträchtigt war (z. B. Amplify 2020; Blainey und Hannay 2020; Brzyska et al. 2021; Department for Education 2021; Domingue et al. 2021; Engzell et al. 2021; Juniper Education 2021; Kogan und Lavertu 2021; Kuhfeld et al. 2020; Maldonado und de Witte 2020; Pier et al. 2021; Rose et al. 2021; Schult et al. 2022; Tomasik et al. 2020). Derartige Nachteile zeigten sich aber nicht in allen Studien bzw. nicht für alle der in den Studien untersuchten Kompetenzdomänen (Depping et al. 2021; Förster et al. 2021; Kuhfeld et al. 2020). Einige Studien deuteten auf Entwicklungsnachteile insbesondere bei jüngeren Schüler_innen hin (Amplify 2020; Juniper Education 2021; Pier et al. 2021; Tomasik et al. 2020). Engzell et al. (2021) berichteten in einer großangelegten niederländischen Studie sogar von Lernverlusten in Mathematik, Lesen und Rechtschreibung in der Primarstufe.

Bisher wenig beleuchtet wurde die Frage, ob bzw. inwieweit sich bildungsbenachteiligende Faktoren auf die Lernentwicklung in Zeiten der Pandemie auswirkten bzw. diese verstärkt haben (Helm et al. 2021). Dies wäre durchaus erwartbar, da primäre Herkunftseffekte (Boudon 1974) sich auf Unterschiede in der Ressourcenausstattung und dem familiären Anregungsgehalt sowie auf Wechselwirkungen mit der Nutzung schulischer Ressourcen zurückführen lassen (z. B. Maaz et al. 2011) und die Schüler_innen durch die Pandemie verstärkt auf Ressourcen und Unterstützung ihres familiären Umfeldes angewiesen waren. So berichteten bildungsbenachteiligte Familien, dass sie ihren Kindern im Homeschooling weniger gut beim schulischen Lernen helfen konnten (Sari et al. 2021). Verschiedene Studien fanden Hinweise darauf, dass sich die für die Entwicklung von Schulleistungen bekannten herkunftsbedingten Schereneffekte während der Corona-Pandemie ausgeweitet haben (Blainey und Hannay 2020; Engzell et al. 2021; Juniper Education 2021; Kogan und Lavertu 2021; Kuhfeld et al. 2020; Maldonado und de Witte 2020; Pier et al. 2021).

Trotz einer Vielzahl an Studien zu Effekten auf Schulleistungen wurde die Frage, wie sich Schüler_innen während der pandemiebedingten Einschränkungen in Bezug auf ihre kognitiven Grundfähigkeiten entwickelt haben, nach unserer Kenntnis bisher noch nicht empirisch behandelt. Auch die Frage, ob es bei der kognitiven Entwicklung von Schüler_innen zu Schereneffekten gekommen ist, die mit den besonderen Lebensbedingungen während der Pandemie in Verbindung gebracht werden können, ist daher offen.

Im Folgenden soll daher zwei Fragestellungen empirisch nachgegangen werden:

1. Wie entwickeln sich die kognitiven Grundfähigkeiten von Schüler_innen im Verlauf eines Schuljahres unter den durch die COVID-19-Pandemie veränderten Lebensbedingungen?
2. Lassen sich für individuelle Merkmale der Kinder sowie für Merkmale ihres häuslichen Umfeldes Zusammenhänge zur Entwicklung ihrer kognitiven Grundfähigkeiten nachweisen, die auf bildungsbenachteiligende Effekte bzw. Schereneffekte hindeuten?

Nach der Metaanalyse von Rindermann (2011) erzielen sechs- bis neunjährige Kinder im deutschsprachigen Raum pro Lebensjahr im Durchschnitt einen Zuwachs von 8,18 IQ-Punkten in ihrer kognitiven Entwicklung. Dies entspricht einer Effektgröße von etwas mehr als einer halben Standardabweichung. Mit Bezug auf Fragestellung 1 wird daher erwartet, dass sich Schüler_innen im Schuljahresverlauf in ihren kognitiven Grundfähigkeiten in einer entsprechenden Größenordnung positiv entwickeln. Zudem wird erwartet, dass sich für individuelle Schülermerkmale und Merkmale des häuslichen Umfeldes Effekte auf die Entwicklung der kognitiven Grundfähigkeiten nachweisen lassen, die Nachteile für bestimmte Schülergruppen belegen und auf Schereneffekte hinweisen (Fragestellung 2).

## Forschungsdesign und Stichprobe

Datengrundlage ist eine im Schuljahr 2020/21 in der dritten Schulstufe an drei österreichischen Volksschulen (Primarstufe) durchgeführte Längsschnittstudie. Zwei der Schulen (mit jeweils zwei Klassen) liegen im Burgenland, sind drei- bis vierzügig (13 bzw. 14 Klassen mit je 20 bis 25 Schüler_innen), haben ein ländliches Einzugsgebiet und weisen eine relativ wenig segregierte Schülerkomposition auf (Lassnigg et al. 2019, S. 115). Die dritte Schule, an der eine Klasse untersucht wurde, ist eine städtische Ganztagsschule in Wien mit sechs Zügen (23 Klassen) und durchschnittlicher sozialer Segregation (ebd.).

Die Paper-Pencil-Befragung der Schüler_innen wurde von den Klassenlehrkräften durchgeführt. Die Lehrkräfte nahmen an einer Online-Schulung zum Einsatz der Fragebogeninstrumente und den zu beachtenden Testinstruktionen teil. Die Bruttostichprobe umfasste 119 Schüler_innen. Davon nahmen 104 Kinder (87,5 %; 48 Mädchen, 56 Jungen) teil, für die eine Zustimmung der Eltern vorlag.

**Abb. 1:**
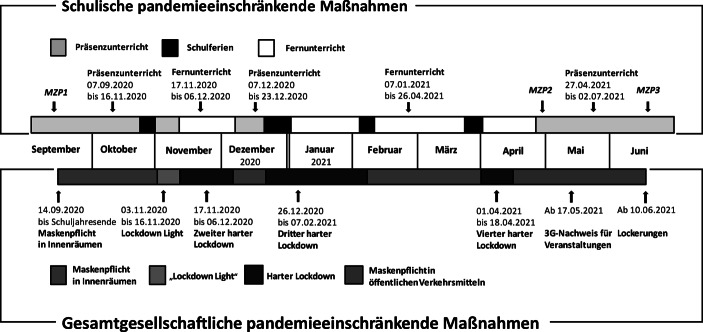
Zeitlicher Verlauf von schulischen und gesamtgesellschaftlichen pandemieeinschränkenden Maßnahmen in Österreich im Schuljahr 2020/21 (07.09.2020–03.07.2021)

Das Untersuchungsdesign (Abb. 1) umfasste drei Messzeitpunkte (MZP) im Schuljahr 2020/21. MZP1 lag zu Schuljahresbeginn im September 2020 und damit neun Wochen vor der ersten pandemiebedingten Schulschließung. Weitere Datenerhebungen erfolgten im Mai 2021 (MZP2) und zum Schuljahresende im Juni 2021 (MZP3), also etwa einen Monat, nachdem die Schulen wieder geöffnet hatten. Von den insgesamt 38 Unterrichtswochen wurden 21 in Präsenz und 17 mit Fernunterricht abgehalten.^1^^,^^2^ Darüber hinaus kam es zu weiteren Unterrichtsausfällen, wenn in Schulklassen Corona-Fälle aufgetreten sind. Die Wochen mit Präsenzunterricht lagen vor allem zu Beginn und Ende des Schuljahres. Neben den Schulschließungen wurden zur Pandemiebekämpfung einschneidende gesamtgesellschaftliche Maßnahmen gesetzt, darunter ein vierzehntägiger Lockdown „Light“ mit Ausgangs‑, Veranstaltungs- und Versammlungsbeschränkungen und drei insgesamt 61 Tage umfassende „harte“ Lockdowns mit ganztägigen Ausgangsbeschränkungen.

### Abhängige Variablen

Zur Erfassung der kognitiven Grundfähigkeiten der Schüler_innen wurden zu beiden MZP die Subtests *Schlussfolgerndes Denken* und *Rechnerisches Denken* des Kognitiven Fähigkeitstests für Kinder (KFT 1–3; Heller und Geisler 1983) eingesetzt. Die Testaufgaben bestehen aus bildlichen Vorlagen, zu denen mündlich eine Testanweisung erteilt wird. Beim Subtest *Schlussfolgerndes Denken* ist anzugeben, „… unter welchem Aspekt sich jeweils vier von fünf vorgegebenen Bildvorlagen gleichen“ (Heller und Geisler 1983, S. 6). Der Subtest *Rechnerisches Denken* erfasst, inwieweit Schüler_innen mit Zahlenbegriffen und mengenmäßigen Vorstellungen umgehen können (ebd.). Jede der beiden Subskalen umfasst 15 Aufgaben. Für richtige Antworten erhalten die Schüler_innen jeweils einen Punkt, für falsche bzw. ausgelassene Antworten null Punkte. Der Subtest *Schlussfolgerndes Denken *weist nach dem Testmanual (ebd.) eine interne Konsistenz von Cronbachs α = 0,56 und über fünf Wochen eine Retest-Reliabilität von r_tt_ = 0,69 auf. Für den Subtest *Rechnerisches Denken *wird eine interne Konsistenz von α = 0,77 und eine Retest-Reliabilität von r_tt_ = 0,65 angegeben.

Die Eindimensionalität der beiden Skalen wurde für den vorliegenden Datensatz für beide MZP mittels explorativer Faktoranalysen geprüft. Die Werte für das Kaiser-Meyer-Olkin-Measure-of-Sampling-Adequacy (KMO) belegen die Angemessenheit der Skalenbildung (*Schlussfolgerndes Denken:* MZP1 = 0,529, MZP3 = 0,546; *Rechnerisches Denken:* MZP1 = 0,745, MZP3 = 0,734). Für das Schlussfolgernde Denken ergibt sich eine interne Konsistenz von α = 0,61 (0,53 ≤ r_it_ ≤ 0,62, MZP1) bzw. α = 0,60 (0,55 ≤ r_it_ ≤ 0,60, MZP3). Für das *Rechnerische Denken* liegt die interne Konsistenz bei α = 0,78 (0,74 ≤ r_it_ ≤ 0,80, MZP1) bzw. α = 0,80 (0,77 ≤ r_it_ ≤ 0,81, MZP3). Die Retest-Reliabilitäten belaufen sich in unserer Stichprobe für das *Schlussfolgernde Denken *auf r_tt_ = 0,67 und für das *Rechnerische Denken *auf r_tt_ = 0,77. Im Niveau entsprechen die ermittelten internen Konsistenzen damit den Werten der Normierungsstichprobe (Heller und Geisler 1983). Die Retest-Reliabilitäten übertreffen in unserer Studie hingegen, trotz des mit neun Monaten deutlich größeren Testabstandes, die im Testmanual berichteten Werte.

### Unabhängige Variablen

Das *Geschlecht* der Kinder (MZP1) wurde dichotom erfasst (0 = *männlich*, 1 = *weiblich*). Das *Alter* der Schüler_innen wurde aus Gründen des Datenschutzes über das Geburtsjahr erhoben. Da nur vier Kinder im Jahr 2013 geboren waren, wurde die Altersvariable dichotomisiert (0 = *Geburtsjahrgänge 2012 und 2013*; 1 = *Geburtsjahrgang 2011*).

Der *Migrationshintergrund* (MZP1) wurde über das Geburtsland des Kindes (*Wurdest du in Österreich geboren?*) und die zu Hause gesprochene Sprache (*Sprichst du in deiner Familie auch eine andere Sprache als Deutsch?*) bestimmt. Wurde das Kind im Ausland geboren und/oder eine andere Sprache als Deutsch zu Hause gesprochen, wurde dies als Hinweis auf einen Migrationshintergrund gewertet (0 = *ohne Migrationshintergrund*; 1 = *mit Migrationshintergrund*).

Die vier weiteren unabhängigen Variablen wurden aus testökonomischen Gründen erst zu MZP2 erhoben. Die dabei eingesetzten Instrumente wurden bezüglich der Anzahl der verwendeten Items und der Antwortkategorien an die Vorgabe bei Kindern ab acht Jahren adaptiert.

Als Indikator für den familiären *schulischen Bildungshintergrund* (MZP2) wurde der höchste Schulabschluss (getrennt für Mutter und Vater) erhoben. Die Antwortkategorien entsprachen den Abschlüssen des österreichischen Schulsystems: 1) *Kein Schulabschluss*, 2) *Pflichtschulabschluss (Hauptschule, Neue Mittelschule),* 3) *Matura (Gymnasium, HTL oder HAK*^3^*) *und 4) *Weiß ich nicht*. Den Familien wird ein höherer Bildungshintergrund zugesprochen, wenn mindestens ein Elternteil die Matura (allgemeine Hochschulreife) erworben hat (0 = *ohne höhere schulische Bildung*, 1 = *mit höherer schulischer Bildung*).

Die *Anzahl der Bücher zu Hause* (MZP2) (Wendt et al. 2017, S. 43) wurde als weiterer Indikator des soziokulturellen Hintergrunds erfasst. Zur leichteren Einschätzung der im Haushalt insgesamt vorhandenen Bücher wurde den Schüler_innen eine Abbildung mit einzelnen Büchern, einem Regalbrett, einem Regal, zwei Regalen und mehreren Regalen mit Büchern vorgelegt.

Um das familiäre bzw. häusliche Umfeld der Kinder in Bezug auf den sozioökonomischen Hintergrund zu charakterisieren, wurden in Anlehnung an PISA 2015 (Mang et al. 2019, S. 28 ff.) zwei Indizes (MZP2) erhoben. Der Index *Familienwohlstand* umfasst vier Items: *Welche der folgenden Dinge gibt es bei dir zu Hause?* 1) *einen Fernseher*, 2) *ein oder mehrere Musikinstrumente*, 3) *einen Rasenmäher* und 4) *ein zweites Auto* (Antwortmöglichkeiten jeweils 0 = *nein*, 1 = *ja*). Sie weisen eine interne Konsistenz von α = 0,79 (0,69 ≤ r_it_ ≤ 0,80) und eine eindimensionale Struktur auf (KMO = 0,621). Der Index *Vorhandensein von digitalen Endgeräten und Internetzugang* wurde über vier Items erfasst: *Welche der folgenden Dinge gibt es bei dir zu Hause?* 1) *Einen Computer (PC, Laptop oder Notebook),* 2) *Internet-Anschluss (W-Lan)*, 3) *Handy mit Internetzugang (z.* *B. Smartphones) *und 4) *Tablet-Computer (z.* *B. iPad, Kindle, usw.)*. Die Items waren mit *ja* (1) oder *nein* (0) zu beantworten und weisen eine angemessen homogene und eindimensionale Struktur auf (KMO = 0,689, α = 0,71, 0,58 ≤ r_it_ ≤ 0,73).

### Fehlende Werte

Von den 104 Schüler_innen der Stichprobe nahmen 87 (83,7 %, MZP1) bzw. 92 (88,5 %, MZP3) Schüler_innen an der Studie teil. 81 Kinder (78,8 %) konnten zu beiden Erhebungszeitpunkten untersucht werden, 23 Kinder (21,2 %) nur zu einem MZP. Fehlende Werte wurden mit *R* (R Core Team 2017) in *RStudio* (RStudio Team 2020) multipel imputiert. Nach den Ergebnissen der Simulationsstudie von Barnes et al. (2006) ist das Verfahren der multiplen Imputation auch bei Stichproben, die kleiner sind als die hier vorliegende, sinnvoll einsetzbar. Die Daten der beiden KFT-Subtests wurden vorab einer Plausibilitätsprüfung unterzogen. Vier Testwerte wurden dabei verworfen, da sie um mindestens eine Standardabweichung niedriger lagen als der zweite für ein Kind vorliegende Testwert. Für die Schätzung der fehlenden Werte wurden die Variablen *Schulzugehörigkeit (idschool), Alter, Migrationshintergrund, Familienwohlstand, Vorhandensein von digitalen Endgeräten und Internetzugang, Anzahl der Bücher zu Hause *und *Schulischer Bildungshintergrund der Eltern *als Hilfsvariablen verwendet. Der Ausfallprozess wurde als *Missing at Random* (MAR) modelliert, da das Muster der fehlenden Werte mit den übrigen Items der Skala zusammenhing (Göthlich 2007, S. 121). Als Verfahren wurden der *Multiple-Imputation-by-Chained-Equations*-Algorithmus (MICE) (van Buuren und Groothuis-Oudshoorn 2011) und der Imputationsalgorithmus *Classification and Regression Trees* (CART) mit 20 Imputationen und 40 Iterationen (Graham et al. 2007) verwendet. Die Imputation führte zu keinen signifikanten Veränderungen in den Mittelwerten oder Streuungen der verwendeten Variablen (Tab. 1 und 2).

**Tab. 1:** Mittelwerte und Streuungen der unabhängigen Variablen

	Imputierte Daten *n* = 104	Unimputierte Daten *n* = 93 (Min.)
	M (SD)	M (SD)
*Geschlecht*	0,52 (0,50)	0,55 (0,50)
*Alter*	0,68 (0,47)	0,69 (0,46)
*Migrationshintergrund*	0,54 (0,50)	0,55 (0,50)
*Schulischer Bildungshintergrund der Eltern*	0,54 (0,50)	0,56 (0,50)
*Anzahl der Bücher zu Hause*	3,13 (1,34)	3,15 (1,37)
*Familienwohlstand*	2,80 (1,22)	2,76 (1,27)
*Vorhandensein von digitalen Endgeräten und Internetzugang*	2,47 (1,30)	2,45 (1,27)

Datengrundlage: Multiple imputierte und unimputierte Daten (*n* = 104 bzw. *n* = 93)

**Tab. 2:** Mittelwerte, Streuungen und Standardfehler des Mittelwerts für die KFT-Subtests *Schlussfolgerndes Denken* und *Rechnerisches Denken* (MZP1 und MZP3)

		Imputierte Daten					Unimputierte Daten				
		M (SD)	SE	t (p)	Levene-Test		M (SD)	SE	t (p)	Levene-Test	
					F (p)	df				F (p)	df
*Schlussfolgerndes Denken*	MZP1	10,91 (1,59)	0,159	0,903 (0,231)	0,015 (0,903)	206	10,87 (1,74)	0,197	0,883 (0,265)	0,022 (0,822)	181
	MZP3	10,91 (1,67)	0,167				10,88 (1,74)	0,175			
*Rechnerisches Denken*	MZP1	11,21 (2,45)	0,240	0,983 (0,343)	0,000 (0,983)	206	11,21 (2,51)	0,285	0,987 (0,384)	0,000 (0,987)	180
	MZP3	11,08 (2,50)	0,245				11,03 (2,63)	0,265			

Datengrundlage: Multiple imputierte und unimputierte (*n* = 104 bzw. *n* = 77) Daten; abhängige t‑Tests und Levene-Tests (zweiseitig) ohne Berücksichtigung des komplexen Stichprobendesigns

Die nachfolgend berichteten Analysen basieren auf imputierten Daten. Im Fall der Mittelwertvergleiche werden auch die Ergebnisse der unimputierten Daten angegeben. Alle inferenzstatistischen Tests wurden, sofern nicht anders angegeben, zweiseitig und mit einer Irrtumswahrscheinlichkeit von *p* < 0,05 ausgeführt. Die Daten der fünf Schulklassen wurden vor der Durchführung der Korrelations- und Regressionsanalysen am Mittelwert der jeweiligen Klasse und dem jeweiligen KFT-Subtest normiert (*Group-Mean-Centering*), um Artefakte aufgrund der Clusterung der Daten zu vermeiden bzw. Effekte auf der Klassenebene zu kontrollieren und Effekte auf Personenebene zuverlässiger untersuchen zu können (Bell et al. 2018). Auf eine Zentrierung der Prädiktoren wurde verzichtet, da dies die Varianz bezüglich der individuell vorliegenden Voraussetzungen reduziert hätte und die Vorteile bzw. Nachteile, die sich für die einzelnen Schüler_innen aufgrund ihrer Hintergrundmerkmale ergeben haben, unterschätzt worden wären. Deskriptivstatistiken und Mittelwertsvergleiche wurden in SPSS 26 berechnet. Die Korrelations- und Regressionsanalysen wurden in Mplus 8.7 (Muthén und Muthén 1998–2022) mit robuster Schätzung der Standardfehler bzw. unter Berücksichtigung der Clusterstruktur der Daten (MLR, *type* *=* *complex*) durchgeführt.

## Ergebnisse

In Tab. 2 werden die Mittelwerte und Streuungen der Testwerte in den KFT-Subtests *Schlussfolgerndes Denken* und *Rechnerisches Denken *berichtet. Für keinen der beiden Subtests ist der über die beiden Messzeitpunkte hinweg erwartete positive Entwicklungstrend über einen Zeitraum von neun Monaten in den Mittelwerten signifikanzstatistisch nachweisbar (Fragestellung 1). Die mittleren Testleistungen stagnieren zu MZP3 auf dem bereits zum MZP1 erreichen Niveau. Auch hinsichtlich der Streuungen unterscheiden sich die Testleistungen der beiden MZP nicht.

Aufgrund dieser Befundlage wird im nächsten Analyseschritt überprüft, ob sich die zum dritten Messzeitpunkt erreichten Testwerte signifikanzstatistisch von den Testwerten abgrenzen lassen, die aufgrund der metaanalytischen Befundlage nach Rindermann (2011) zu erwarten gewesen wären. Für den hier vorliegenden Untersuchungszeitraum von neun Monaten ist dies ein Entwicklungsfortschritt, der einer Effektgröße von rund d = 0,40 entspricht. Die Eingruppen-t-Tests ergeben in beiden Fällen ein signifikantes Ergebnis (*Schlussfolgerndes Denken*: t = −3,75**, *p* < 0,001; *Rechnerisches Denken*: t = −4,00**, *p* < 0,001; t‑Tests ohne Berücksichtigung des komplexen Stichprobendesigns).

Tab. 3 stellt die bivariaten Korrelationen zwischen den betrachteten individuellen Merkmalen bzw. den Merkmalen des häuslichen Umfelds und den Testleistungen im *Schlussfolgernden Denken* und *Rechnerischen Denken* zu MZP1 und MZP3 dar. Darüber hinaus sind die Partialkorrelationen mit den Testwerten zu MZP3 unter Kontrolle der Testwerte zu MZP1 angegeben. Durch die statistische Kontrolle der Ausgangsleistungen wird die kognitive Entwicklung, die sich zwischen den beiden MZP vollzogen hat, in den Blick genommen. Die Partialkorrelationen weisen darauf hin, dass sich Kinder aus Familien, in denen mindestens ein Elternteil über die allgemeine Hochschulreife verfügt, in Bezug auf das *Schlussfolgernde Denken* und das *Rechnerische Denken* signifikant günstiger entwickeln als Kinder, bei denen dies nicht der Fall ist (r._MZP1_ = 0,355** bzw. 0,319**). Dies gilt auch für Kinder, die in einem häuslichen Umfeld aufwachsen, das in Bezug auf digitale Endgeräte bzw. einen Internetzugang besser ausgestattet ist (r._MZP1_ = 0,425** bzw. 0,306*). Zudem entwickeln sich Kinder aus Haushalten, in denen eine höhere Anzahl an Büchern vorhanden ist, im Rechnerischen Denken etwas positiver (r._MZP1_ = 0,131**).

**Tab. 3:** Bivariate Korrelationen und Partialkorrelationen der KFT-Subtests *Schlussfolgerndes Denken* und* Rechnerisches Denken* mit individuellen Schülermerkmalen bzw. Merkmalen des häuslichen Umfeldes

	KFT1–3 Schlussfolgerndes Denken			KFT1–3 Rechnerisches Denken		
	MZP1	MZP3	MZP3 unter Kontrolle MZP1	MZP1	MZP3	MZP3 unter Kontrolle MZP1
*Geschlecht*	−0,026	0,017	0,045	−0,001	0,001	0,003
	(0,793)	(0,886)	(0,748)	(0,983)	(0,988)	(0,977)
*Alter*	0,302**	0,365**	0,219^+^	0,157*	0,189	0,106
	(0,002)	(0,002)	(0,073)	(0,010)	(0,162)	(0,496)
*Migrationshintergrund*	0,005	0,006	0,004	0,081	0,110^+^	0,077
	(0,957)	(0,954)	(0,974)	(0,414)	(0,069)	(0,343)
*Schulischer Bildungshintergrund der Eltern*	0,024	0,281*	0,355**	0,415**	0,522**	0,319**
	(0,871)	(0,003)	(< 0,001)	(< 0,001)	(< 0,001)	(< 0,001)
*Anzahl der Bücher zu Hause*	0,005	0,058	0,073	0,414**	0,420**	0,131**
	(0,967)	(0,704)	(0,634)	(< 0,001)	(0,000)	(0,002)
*Familienwohlstand*	0,124^+^	0,110	0,036	0,268^+^	0,285*	0,111
	(0,065)	(0,100)	(0,499)	(0,071)	(0,016)	(0,198)
*Vorhandensein von digitalen Endgeräten und Internetzugang*	0,457**	0,622**	0,425**	0,179	0,316**	0,306**
	(< 0,001)	(< 0,001)	(< 0,001)	(0,284)	(0,034)	(< 0,001)

Datengrundlage: Multiple imputierte Daten. Korrelation nach Pearson (in Klammern: *p*-Werte), Partialkorrelationen unter Kontrolle der Ausgangsleistungen zu MZP1 (jeweils am Klassenmittelwert normiert). Kodierung der Variablen: Geschlecht: 0 = männlich, 1 = weiblich; Migrationshintergrund: 0 = ohne Migrationshintergrund, 1 = mit Migrationshintergrund; schulischer Bildungshintergrund der Eltern: 0 = kein Elternteil mit allgemeiner Hochschulreife, 1 = mindestens ein Elternteil mit allgemeiner Hochschulreife. Berechnungen mit Mplus 8.7 unter Verwendung robust geschätzter Standardfehler (MLR, type = complex)

**p < 0,01; *p < 0,05; ^+^*p* < 0,10 (zweiseitig)

Für die multiplen Regressionsanalysen (Tab. 4) wurden alle oben genannten individuellen Merkmale der Schüler_innen bzw. Merkmale des häuslichen Umfeldes als Prädiktoren herangezogen. Abhängige Variablen sind die Testleistungen im *Schlussfolgernden Denken* bzw. im *Rechnerischen Denken* zu MZP3. In den Modellen wird die jeweilige Ausgangsleistung zu MZP1 kontrolliert, um die Entwicklung zwischen den beiden Erhebungszeitpunkten abzubilden. Für die Entwicklung im *Schlussfolgernden Denken* und im *Rechnerischen Denken* erweisen sich neben der jeweiligen Ausgangsleistung zu MZP1 im multiplen Regressionsmodell zwei Merkmale als statistisch signifikante Prädiktoren: Schüler_innen, deren Eltern einen höheren schulischen Bildungshintergrund haben (β = 0,175*, *p* = 0,011 bzw. β = 0,163**, *p* = 0,004) und in deren Familien eine bessere Ausstattung mit digitalen Endgeräten bzw. einem Internetzugang gegeben ist (β = 0,332**, *p* = 0,004 bzw. β = 0,106**, *p* < 0,001), entwickeln sich im Schuljahresverlauf günstiger. Für alle anderen Prädiktoren ergeben sich keine Hinweise auf problematische Schereneffekte in der kognitiven Entwicklung.

**Tab. 4:** Regressionsmodell zur Vorhersage des *Schlussfolgernden Denkens* und des *Rechnerischen Denkens*

	KFT1–3 Schlussfolgerndes Denken			KFT1–3 Rechnerisches Denken		
	B	SE	β	B	SE	β
*Ausgangsleistung zu MZP1*	0,516**	0,126	0,482**	0,742**	0,081	0,714**
*Geschlecht*	0,190	0,258	0,055	0,057	0,265	0,012
*Alter*	0,395	0,302	0,107	0,196	0,375	0,039
*Migrationshintergrund*	−0,274	0,238	−0,080	0,098	0,145	0,021
*Schulischer Bildungshintergrund der Eltern*	0,599*	0,273	0,175*	0,773**	0,263	0,163**
*Anzahl der Bücher zu Hause*	−0,038	0,089	−0,030	0,101	0,101	0,057
*Familienwohlstand*	−0,032	0,044	−0,023	0,068	0,093	0,035
*Vorhandensein von digitalen Endgeräten und Internetzugang*	0,441**	0,128	0,332**	0,194**	0,040	0,106**
*R (R*^*2*^*)*	**0,782 (0,612)**			**0,873 (0,763)**		

Datengrundlage: Multiple imputierte Daten; Werte der AV am Klassenmittelwert normiert. Kodierung der Variablen: Geschlecht: 0 = männlich, 1 = weiblich; Migrationshintergrund: 0 = ohne Migrationshintergrund, 1 = mit Migrationshintergrund; schulischer Bildungshintergrund der Eltern: 0 = kein Elternteil mit allgemeiner Hochschulreife, 1 = mindestens ein Elternteil mit allgemeiner Hochschulreife. Berechnungen mit Mplus 8.7 unter Verwendung robust geschätzter Standardfehler (MLR, type = complex)

Unstandardisierte (*B*) bzw. standardisierte (*β*) Regressionskoeffizienten, *SE* Standardfehler (robust)

**p < 0,01; *p < 0,05; ^+^*p* < 0,10 (zweiseitig)

## Zusammenfassung und Diskussion

Für den Bereich schulischer Leistungen liegen national wie international empirische Studien vor, die darauf hinweisen, dass die pandemieeinschränkenden Maßnahmen nachteilige Effekte auf die Entwicklung von Schüler_innen hatten. Zudem wurden für Schulleistungen Schereneffekte zuungunsten bildungsbenachteiligter Schüler_innen beschrieben (Helm et al. 2021). Unklar ist bisher, ob und inwieweit sich die pandemiebedingt veränderten Lebensbedingungen auch auf ihre generelle kognitive Entwicklung niedergeschlagen haben. Im vorliegenden Beitrag wurden dazu Daten von 104 Kindern der dritten Schulstufe dreier österreichischer Primarschulen analysiert. Die kognitiven Grundfähigkeiten wurden mit den beiden Subtests *Schlussfolgerndes Denken* und *Rechnerisches Denken* des Kognitiven Fähigkeitstests 1–3 (KFT 1–3, Heller und Geisler 1983) erfasst. Die Datenerhebung erfolgte im Abstand von neun Monaten zu Beginn und am Ende des Schuljahres 2020/21, in dem es in Österreich zu gravierenden pandemieeinschränkenden Maßnahmen wie z. B. Kontakt- und Ausgangsbeschränkungen oder Schulschließungen (s. Abb. 1) kam. Nach den metanalytischen Befunden von Rindermann (2011) wäre über einen – wie in unserer Studie vorliegenden – Untersuchungszeitraum von neun Monaten eine insgesamt positive Entwicklung der kognitiven Grundfähigkeiten der Kinder mit einer Effektgröße von etwa 0,40 Standardabweichungen zu erwarten gewesen.

Tatsächlich konnte sich die Gruppe der hier untersuchten Schüler_innen insgesamt in keinem der beiden Subtests signifikant verbessern, sondern verharrte im Mittel auf dem bereits zum Schuljahresbeginn erreichten kognitiven Fähigkeitsniveau. Diese Befundlage unterstützt die Annahme, dass für die Gesamtstichprobe in dem von pandemieeinschränkenden Maßnahmen geprägten Untersuchungszeitraum Entwicklungsbedingungen vorlagen, die einer altersgemäßen positiven Weiterentwicklung der kognitiven Grundfähigkeiten entgegenstanden. Bemerkenswert ist zudem, dass sich auch die Leistungsstreuungen in dieser Zeit nicht verändert haben. Die Heterogenität in den kognitiven Grundfähigkeiten hat sich demnach in der untersuchten Stichprobe trotz der pandemiebedingt veränderten Lebensbedingungen nicht systematisch ausgeweitet.

Allerdings weisen die Ergebnisse der Korrelations- und Regressionsanalysen auf differentielle Entwicklungsverläufe in Abhängigkeit von häuslichen Merkmalen hin. Unter Kontrolle der Ausgangsfähigkeit entwickelten sich Schüler_innen, deren Eltern einen höheren Bildungshintergrund aufwiesen bzw. die für ihr häusliches Umfeld eine höhere Verfügbarkeit von digitalen Endgeräten und Internet angaben, hinsichtlich ihrer Fähigkeiten zum Schlussfolgernden Denken und Rechnerischen Denken günstiger.

Insgesamt deuten die berichteten Befunde damit darauf hin, dass es bei der Entwicklung kognitiver Grundfähigkeiten zu Schereneffekten gekommen ist. Mit Blick auf den allgemein ausbleibenden Anstieg in den mittleren Leistungen und den über die MZP homogenen Streuungen ist diese Befundlage durchaus überraschend, da vor diesem Hintergrund der Nachweis von Schereneffekten bedeutet, dass sich einige Kinder in ihrer kognitiven Leistungsfähigkeit weiterentwickelt haben, während es für andere in dieser Hinsicht zu Einbußen gekommen zu sein scheint.

Bei der Interpretation der hier vorgestellten Befunde ist eine Reihe von Limitationen zu berücksichtigen. Die zugrundeliegende Studie umfasst lediglich 104 Kinder aus fünf Klassen der dritten Schulstufe aus drei österreichischen Volksschulen (Primarstufe). Inwieweit sich die für diesen Kontext ermittelten Ergebnisse verallgemeinern lassen, muss offenbleiben und bedarf weiterer Forschungsbemühungen. Es liegen keine Daten einer Kontrollgruppe vor. Eine solche ließ sich bisher nicht realisieren, da seit Untersuchungsbeginn alle Schulkinder von pandemieeinschränkenden Maßnahmen betroffen waren. Auch geeignete Vergleichsdaten von österreichischen Kindern der dritten Schulstufe, die vor dem Einsetzen der Corona-Pandemie mit einem vergleichbaren Instrumentarium und längsschnittlichen Design erhoben wurden, stehen nicht zur Verfügung. Daher wurden metaanalytische Befunde zu der für diese Altersgruppe im deutschsprachigen Raum für einen Zeitraum von neun Monaten zu erwartenden Intelligenzentwicklung als Referenz herangezogen. Unsere Analysen beruhen zudem nur auf zwei der vier KFT-Subtests. Die Höhe der Reliabilitäten der beiden Testverfahren (Cronbachs α: 0,61/0,60 bzw. 0,78/0,80) können zumindest einen Teil des nicht zu beobachtenden Entwicklungsfortschritts erklären. Darüber hinaus wurde im Rahmen unserer Studie keine Elternbefragung durchgeführt. Entsprechend basieren die individuellen Schülermerkmale bzw. häuslichen Merkmale auf selbstberichteten Angaben von Schulkindern. Die aus der Literatur entlehnten Instrumente mussten für die vorliegende Altersstufe gekürzt und vereinfacht werden.

Trotz der genannten Einschränkungen liefern die hier berichteten Befunde aus unserer Sicht wichtige Hinweise dahingehend, dass die pandemiebedingten Veränderungen der Lebenswelt von Schüler_innen der Primarstufe, wie sie Kontaktbeschränkungen, Schulschließungen bzw. Fernunterricht oder Restriktionen des Freizeitangebotes darstellten, ein weniger günstiges Umfeld für die Weiterentwicklung ihrer kognitiven Grundfähigkeiten geboten haben könnten. Für Kinder, die im Vergleich zu ihren Mitschüler_innen bildungsbenachteiligende Merkmale aufweisen, deuten die hier berichteten Befunde zudem an, dass es in dem durch die Pandemie geprägten Schuljahr zu Entwicklungsnachteilen gekommen ist. Ob sich bestehende Schereneffekte im Zuge der Pandemie verschärft haben, lässt sich dabei durch das Fehlen einer geeigneten Vergleichsgruppe vor Beginn der Pandemie nicht klären. Die längsschnittliche Homogenität der Streuungen der kognitiven Testleistungen spricht im vorliegenden Fall aber gegen diese Vermutung.

Ob sich die hier für den Bereich der allgemeinen kognitiven Grundfähigkeit berichteten Befunde verallgemeinern lassen, muss die weitere Forschung zeigen. Hier wären Sekundäranalysen von empirischen Daten wünschenswert, die in den Schuljahren 2020/21 bzw. 2021/22 Indikatoren der kognitiven Grundfähigkeit längsschnittlich (mit)erfasst haben, wie dies in entsprechend angelegten Schulleistungsstudien üblich ist. Damit ließe sich ein wichtiger Beitrag für die international bedeutsame Forschungsfrage leisten, ob bzw. in welchen Bereichen in der Zeit pandemiebedingter Einschränkungen Kindern und Jugendlichen Entwicklungsnachteile entstanden sind, um diesen ggfs. systematisch entgegenwirken zu können. Dabei wird es nicht genügen, einfach nur die vorpandemischen Lebensumstände wiederherzustellen. Vielmehr sind gezielte und konzertierte Programme angezeigt, um Entwicklungsanreize zu setzen und die durch die Kinder und Jugendlichen ggfs. erlittenen Nachteile so weit wie möglich auszugleichen.

## Footnotes

1 BMBWF GZ 2020‑0.748.656.

2 BMBWF GZ 2021‑0.202.824.

3 Eine Höhere Technische Lehranstalt (HTL) ist in Österreich eine berufsbildende höhere Schule mit technischen, gewerblichen und kunstgewerblichen Ausbildungsschwerpunkten. Die Handelsakademie (HAK) ist eine berufsbildende höhere Schule (BHS), deren Schwerpunkt eine kaufmännische und betriebswirtschaftliche Ausbildung ist. Die Absolvent_innen schließen mit der Matura ab und sind damit studienberechtigt.
